# BMC Biochemistry Reviewer Acknowledgement, 2015

**DOI:** 10.1186/s12858-016-0058-9

**Published:** 2016-02-10

**Authors:** Guangde Tu

**Affiliations:** Room 1504–1505 Financial Plaza, No. 333 Jiujiang Road, Huangpu District, Shanghai 200001 China

## Abstract

The editors of BMC Biochemistry would like to thank all our reviewers who have contributed their time to the journal in Volume 16 (2015).

**Basir Ahmad**

India

**Manuel Aureliano**

Portugal

**Petity Balaji**

India

**Francisco Belda**

Spain

**Kathryn Bollinger**

USA

**Gloria Borgstahl**

USA

**Nanette Boyle**

USA

**Inka Brockhausen**

Canada

**Nediljko Budisa**

Germany

**Chris Bystroff**

USA

**Bruno Canard**

France

**Virginia Carbone**

Italy

**Michael Carty**

Ireland

**Gopal Chakrabarti**

India

**Anupam Chakravarty**

USA

**Angela Chambery**

Italy

**Yabin Cheng**

China

**Peter Chivers**

United Kingdom

**Kyung-Chul Choi**

Republic of Korea

**Stefano Ciurli**

Italy

**Paul Cobine**

USA

**Rachel Codd**

Australia

**Gwenaelle Conseil**

Canada

**Roberto Consonni**

Italy

**Concetta De Santi**

Norway

**Laurent Desaubry**

France

**Andrew Doig**

United Kingdom

**Robert Domaoal**

USA

**Nicolas Doucet**

Canada

**Charles Evans**

USA

**Andreas H Ewald**

Germany

**Monique Floer**

USA

**Christian Frezza**

United Kingdom

**Yu Gao**

USA

**Takuya Genda**

Japan

**Manik Ghosh**

India

**Andrew Grant**

United Kingdom

**Dietmar Haltrich**

Austria

**Jaeseok Han**

Republic of Korea

**Hartmut Jaeschke**

USA

**Dafydd Jones**

United Kingdom

**Rameshwar Kadam**

USA

**Hisato Kawakami**

Japan

**Stephen Keyse**

United Kingdom

**Sunghoon Kim**

Republic of Korea

**Ruth Kluck**

Australia

**Manfred Konrad**

Germany

**Jan Konvalinka**

Czech Republic

**Barbara Krajewska**

Poland

**Sandeep Kumar**

USA

**Po-Lin Kuo**

Taiwan

**Konstantinos Lefkimmiatis**

United Kingdom

**Elaine Leslie**

Canada

**Christina Leslie**

USA

**Stephen Levene**

USA

**Gail Lewis Phillips**

USA

**Zheng Li**

China

**Chung-Dar Lu**

USA

**Richard Mains**

USA

**Elena Marcello**

Italy

**Assen Marintchev**

USA

**Timothy McKinsey**

USA

**Amy Medlock**

USA

**Gustavo Menezes**

Brazil

**Hua Naranmandura**

China

**Heinz Nasheuer**

Ireland

**Helena Nevalainen**

Australia

**Sangtaek Oh**

Republic of Korea

**Caryn Outten**

USA

**Kelli Palmer**

USA

**Ji-Cheng Pan**

China

**Virendra Pandey**

USA

**Giulia Piaggio**

Italy

**Gianluca Picariello**

Italy

**Ekaterina Pletneva**

USA

**Mohamad Rafi**

Indonesia

**Sanghamitra Raha**

India

**Anita Rajmane**

India

**Vickram Ramkumar**

USA

**Larry Reitzer**

USA

**Thore Rohwerder**

Germany

**Arnold Ruoho**

USA

**Brandy Russell**

USA

**Neelam Sangwan**

India

**Silvia C. Finnemann**

USA

**Vern L. Schramm**

USA

**Suparna Sengupta**

India

**Mehdi Shakibaei**

Germany

**William Sheffield**

Canada

**Robert Silverman**

USA

**Ladaslav Sodek**

Brazil

**Brendan Stamper**

USA

**Reinhard Sterner**

Germany

**Ildiko Szabo**

Italy

**Yuko Tsutsui**

USA

**Kuruthihalli S. Vishwanatha**

USA

**Suning Wang**

USA

**Irene Weber**

USA

**Tomasz Wegierski**

Poland

**Sean Weise**

USA

**Marek Wesołowski**

Poland

**Birgitta Wöhrl**

Germany

**Dieter Wolf**

USA

**Jiao Yang**

USA

**Zhaogang Yang**

USA

**Yan Zhang**

China

**Liangjun Zhao**

USA

